# Diversity of West Nile and Usutu virus strains in mosquitoes at an international airport in Austria

**DOI:** 10.1111/tbed.14198

**Published:** 2021-07-08

**Authors:** Karin Bakran‐Lebl, Jeremy V. Camp, Jolanta Kolodziejek, Pia Weidinger, Peter Hufnagl, Adriana Cabal Rosel, Andreas Zwickelstorfer, Franz Allerberger, Norbert Nowotny

**Affiliations:** ^1^ Austrian Agency for Health and Food Safety Institute for Medical Microbiology and Hygiene Vienna Austria; ^2^ Institute of Virology University of Veterinary Medicine Vienna Vienna Austria; ^3^ Airside Operations Vienna International Airport Wien‐Flughafen Austria; ^4^ Department of Basic Medical Sciences College of Medicine Mohammed Bin Rashid University of Medicine and Health Sciences Dubai Healthcare City Dubai United Arab Emirates

**Keywords:** airport, Austria, *Culex pipiens*, mosquito‐borne diseases, Usutu virus, virus monitoring, West Nile virus

## Abstract

Increased globalization and international transportation have resulted in the inadvertent introduction of exotic mosquitoes and new mosquito‐borne diseases. International airports are among the possible points of entry for mosquitoes and their pathogens. We established a mosquito and mosquito‐borne diseases monitoring programme at the largest international airport in Austria and report the results for the first two years, 2018 and 2019. This included weekly monitoring and sampling of adult mosquitoes, and screening them for the presence of viral nucleic acids by standard molecular diagnostic techniques. Additionally, we surveyed the avian community at the airport, as birds are potentially amplifying hosts. In 2018, West Nile virus (WNV) was detected in 14 pools and Usutu virus (USUV) was detected in another 14 pools of mosquitoes (minimum infection rate [MIR] of 6.8 for each virus). Of these 28 pools, 26 consisted of female *Culex pipiens/torrentium*, and two contained male *Culex* sp. mosquitoes. *Cx. pipiens/torrentium* mosquitoes were the most frequently captured mosquito species at the airport. The detected WNV strains belonged to five sub‐clusters within the sub‐lineage 2d‐1, and all detected USUV strains were grouped to at least seven sub‐clusters among the cluster Europe 2; all strains were previously shown to be endemic in Austria. In 2019, all mosquito pools were negative for any viral nucleic acids tested. Our study suggests that airports may serve as foci of arbovirus activity, particularly during epidemic years, and should be considered when designing mosquito control and arbovirus monitoring programmes.

## 1 INTRODUCTION

Over the past few decades, there has been an increased incidence of (re‐)emerging mosquito‐borne diseases in Europe (Barzon, 2018; Gratz, 1999; Medlock et al., 2012). While globalization facilitates the accidental spreading of mosquitoes and mosquito‐borne pathogens via the increased transportation of goods and people, environmental change and global warming could allow introduced exotic mosquito species and pathogens to become established in previously unsuitable areas (Brugueras et al., 2020; El‐Sayed & Kamel, 2020; Fischer et al., 2020; Medlock et al., 2012; Semenza & Suk, 2018). Viruses originating from tropical regions in sub‐Saharan Africa, like West Nile virus (WNV; Marcantonio et al., 2015), Usutu virus (USUV; Vilibic‐Cavlek et al., 2020), or Chikungunya virus (CHIKV; Amraoui & Failloux, 2016), are meanwhile increasingly occurring in Europe. Some of those pathogens can only spread because the appropriate mosquito vector was introduced previously. For example, the spread of invasive Asian tiger mosquitoes (*Aedes albopictus*) in Europe has been associated with outbreaks of Dengue virus (DENV) in France and Croatia in 2010, and CHIKV in Italy in 2007 and France in 2010 (Medlock et al., 2012). Other newly emerging pathogens can be transmitted by native mosquitoes. WNV and USUV, which are now well established and widely spread across Europe, were likely introduced by migratory birds and are readily transmitted by competent native mosquito vectors of the *Culex pipiens* complex (Brugman et al., 2018; Camp & Nowotny, 2020; Hubálek, 2008).

Possible points of entry for mosquitoes and their pathogens are airports. Although the importation of exotic mosquitoes via air travel seems to be a rare event, importations have been repeatedly reported in recent years (Ibañez‐Justicia et al., 2017; Ibáñez‐Justicia et al., 2020; Scholte et al., 2014). Apparently, an increasing volume of air transportations elevates the risk of accidental introduction of exotic mosquito species. It is therefore recommended by the World Health Organization (WHO) and the European Centre for Disease Prevention and Control (ECDC) to carry out mosquito monitoring programmes at airports (ECDC, 2012; WHO, 2016).

There are two main ways in which mosquito‐borne diseases can be introduced via air travel. First, infected mosquitoes could be transported via the aircraft. For example, malaria cases have been documented in and near international airports among persons who have not recently travelled to areas where the disease was endemic (Gratz et al., 2000). These ‘airport malaria’ cases are caused by malaria‐infected mosquitoes travelling by aircraft from a country where malaria is endemic to a country in which malaria is usually not found, where the mosquito then bites a person in or nearby the airport and transmits the malaria parasite. Those are, however, rare events occurring mainly at airports with high connection frequencies to the main endemic areas in sub‐Saharan Africa (Guillet et al., 1998). This transmission route is of course not limited to malaria, as other mosquito‐borne diseases seem to spread accordingly (e.g. ‘airport Dengue’; Whelan et al., 2012). Airplane disinsection is an important method to prevent or at least reduce the risk of this transmission route (Gratz et al., 2000). The second major pathway of introducing novel mosquito‐borne diseases via aircraft is the transportation of already infected persons (Feng et al., 2019). Upon arriving at their destination, infected passengers may be bitten by native mosquitoes that further spread the pathogens, provided these mosquitoes are competent vectors (Quam et al., 2015). Autochthonous transmission of arboviruses originating from infected travellers appears to be more frequent, with several examples of DENV infection (Franco et al., 2015; Kutsuna et al., 2015; Marchand et al., 2013) and CHIKV infection (Calba et al., 2017; Delisle et al., 2015) arising across Europe in patients with no travel history.

In this study, we present the results of a mosquito‐monitoring programme at a large international airport in Austria. Our monitoring scheme was designed to detect the importation of non‐native mosquito species and non‐native arboviruses. As some zoonotic arboviruses use birds as amplifying hosts, we additionally surveyed the avian community at the airport and tested birds killed by plane strikes for the presence of arbovirus nucleic acids. The goal was to implement a monitoring programme, in addition to the ongoing national and regional monitoring programmes, and to assess the utility of such a programme to monitor the mosquito population and arbovirus activity with a specific focus on detecting exotic mosquitoes and/or viruses.

## 2 MATERIALS AND METHODS

### 2.1 Study site

A mosquito monitoring programme was established at the Vienna International Airport, near Vienna, Austria (48.111°N, 16.569°E, 183 m above sea level). The airport lies on the north‐western edge of the Pannonian biogeographic region, which is characterized by a humid continental climate. The airport is the largest in Austria, connecting to more than 170 international destinations and transporting over 25 million passengers each year.

The sampling took place in a green courtyard, approximately 70 m from the airport's movement area. A small pond (∼0.5 m^3^) within this courtyard was stocked with non‐native fish for a brief time in 2018. When the courtyard was no longer used by the employees, the fish were removed; however, the water remained in the pond until the end of July 2019, when the pond was filled up with soil. Moreover, the vegetation at this site was no longer maintained on a regular basis in 2019.

### 2.2 Mosquito trapping and identification

Weekly sampling of adult mosquitoes was performed using a BG‐Sentinel 2 mosquito trap (Biogents AG, Regensburg, Germany) equipped with an additional CO_2_ release and a specific lure (BG‐Sweetscent). The trap was operated continuously for one week, and the contents were collected weekly from 13 June 2018 to 31 October 2018 and from 2 May 2019 to 30 October 2019. There were thus 18 collection events (trap‐weeks) in 2018 and 26 trap‐weeks in 2019.

Female mosquitoes were identified to species level by morphological characteristics using the keys of Becker et al. (2010) and Gunay et al. (2018), with the exception of mosquitoes from the *Anopheles maculipennis* complex, and the species *Culex pipiens/torrentium* and *Aedes cinerus/geminus*, as those species cannot be reliably distinguished based on morphological characteristics alone. In Austria, the *Cx. pipiens* complex is comprised of *Cx. pipiens* form *pipiens* Linnaeus and *Cx. pipiens* form *molestus* Forskal (Zittra et al., 2016). Male mosquitoes were identified to genus level. Single voucher specimens were identified by molecular barcoding of a portion of the *cytochrome c oxidase I* (*cox1*) gene as previously described (Camp et al., 2019) using primers VF1d and VR1d (Ivanova et al., 2007) and aligning sequences to an in‐house database. The *cox1* genomic region includes a point mutation useful for distinguishing *Cx. pipiens* f. *pipiens* from *Cx. pipiens* f. *molestus* (Shaikevich, 2007).

### 2.3 Testing for flaviviruses

Following morphological identification, mosquitoes were pooled according to capture week, species, and sex, with a maximum number of 20 individuals per sample, and then stored for further analysis at –80°C. Mosquito pools were homogenized in Dulbecco's Modified Eagle's Medium (DMEM, Gibco, Dublin, Ireland) using 2.8‐mm ceramic beads (Bertin Technologies, Montigny‐le‐Bretonneux, France) in a TissueLyser II (Qiagen, Hilden, Germany). After centrifugation, 200 μL of each homogenized pool's supernatant was processed by automated nucleic acid extraction employing a QIAamp Viral RNA Mini Kit (Qiagen, Hilden, Germany) in a QIAcube HT extraction device (Qiagen, Hilden, Germany) according to the manufacturer's instructions. The nucleic acid extracts were screened by WNV (lineage 1+2)‐ and USUV‐specific reverse transcription quantitative (RT‐q)PCRs as described previously (Kolodziejek et al., 2014; Weissenböck et al., 2013). To confirm the possible presence of these two viruses, as well as to detect other flaviviruses (e.g. Bagaza virus, DENV, Barkedji virus or mosquito‐specific flaviviruses), a universal flavivirus RT‐PCR within the *non‐structural protein 5* (*NS5*) genomic region was performed with a previously published, degenerated primer pair (Flavi all S / Flavi all AS2) (Patel et al., 2013). Although this assay was designed as RT‐qPCR, it was performed as a conventional RT‐PCR (without probe) using OneStep RT‐PCR Kit (Qiagen, Hilden, Germany). To determine the identities of the detected viruses and their genetic variants, PCR products (amplicon lengths dependent on the specific virus, app. 260 bp) were subsequently subjected to Sanger sequencing (Eurofins Genomics, Ebersberg, Germany).

To estimate the proportion of infected mosquitoes we calculated the MIR for unequal pool sizes as described previously (Biggerstaff, 2008). The MIR specifies the ratio of the number of positive pools to the total number of mosquitoes tested per 1000 individuals. Data analysis was conducted in R (R Core Team, 2020).

### 2.4 Phylogenetic analysis

In order to compare WNV and USUV sequences to those previously published from Austria, we used an RT‐PCR assay that is universal for the Japanese encephalitis virus complex (Weissenböck et al., 2002), targeting an approximately 750‐bp long genome fragment within the *NS5* and 3′ untranslated region (3′UTR). However, to increase PCR specificity and to improve sequencing results, two virus‐specific primer pairs were designed in comparable gene regions, amplifying 870‐bp and 735‐bp long PCR products, respectively. For this purpose, WNV strain Goshawk‐Hungary/04 (GenBank acc. no. DQ116961) and USUV strain Vienna 2001 (GenBank acc. no. AY453411) served as reference sequences; primers were designed using the Primer Designer programme (Scientific & Educational Software) (Table 1). Following RT‐PCR with the designed primers, amplicons of the appropriate size were sequenced in both directions as described above.

**TABLE 1:** Primers and probes established or modified for this study

Assay name	Oligo name/ direction	Oligonucleotide sequence 5′‐3′	Genome position	Reference sequence	Amplicon size (bp)
WNV‐specific RT‐PCR	WNV_F	TCGCAGTCTGGAACAGAGTG	10127‐10146	DQ116961	870
	WNV_R	GCTGGTTGTGCAGAGCAGAA	10977‐10996		
USUV‐specific RT‐PCR	USUV_F	AGTGCATGCCACAGGTGAAT	10086‐10105	AY453411	735
	USUV_R	AGTTCGCATCACCGTCTGTT	10801‐10820		
CVOV/BATV‐specific RT‐qPCR	CVOV_F	GATGTCGCTGCTAACACCAG	90‐109	KJ542624	171
	CVOV_R	GTTAAGCGTAACCTCCCATTCACT	260‐237		
	CVOV_P	ACACCACTGGGCTTAGTTATGAC^a^	157‐179		
TAHV‐specific RT‐qPCR	TAHV_F	CTGGGTTGTGCCCAGGTT	918‐935	HM036209	70
	TAHV_R	GAAGCTGGCCCTTTGGATTT	968‐987		
	TAHV_P	TCTCAGGGCTGCAAGAGTCATGTG^a^	943‐966		
SINV‐specific RT‐qPCR^b^	SINV_F	GGTTCCTACCACAGCGACGAT	227‐247	M69205	75
	SINV_R	TG**R**TACTGGTGCTCGGAAAACA	280‐301		
	SINV_P	TTGGACATAGGCAGCGCA^a^	249‐266		

Abbreviations: F, forward primer; R, reverse primer; P, probe. ^a^Probes were labelled at the 5′‐end with 6‐carboxyfluorescein (FAM) and the 3′‐end with tetramethyl‐6‐carboxyrhodamine (TAMRA). ^b^ Sane et al. (2012); modified nucleotide in bold.

For the phylogenetic analysis, sequences determined in this study were aligned with selected WNV and USUV sequences (50 each) from GenBank using ClustalW multiple alignments in BioEdit Alignment Editor version 7.0.9.0. Phylogenetic trees were created with the MEGA X programme (Kumar et al., 2018) by application of the neighbour‐joining method and p‐distance algorithm with 1000 replicates of bootstrap resampling analysis each. To construct the phylogenetic trees, sequences had to be shortened [to 690 nucleotides (nt) for WNV and to 695 nt for USUV] to account for differences in the 3′UTR.

All WNV and USUV sequences generated in this study were uploaded to the NCBI database with the following accession numbers: MW160840‐MW160849 and MW160850‐MW160861, respectively.

### 2.5 Other molecular investigations

All mosquito extracts were additionally tested by virus‐specific RT‐qPCRs for two endemic orthobunyaviruses (family *Peribunyaviridae*): Batai orthobunyavirus (Calovo virus, CVOV) and Tahyna orthobunyavirus (TAHV), using in‐house primers developed via Primer Express Software version 3.0 (Applied Biosystems, Foster City, CA, USA) based on CVOV strain 134, segment S (GenBank acc. no. KJ542624) and TAHV isolate Prototype ‘92’ Bardos, segment M (GenBank acc. no. HM036209), respectively. Mosquito RNA extracts were also tested for the presence of two alphaviruses: CHIKV and Sindbis virus (SINV). While CHIKV RT‐qPCR (primer set 874F/961R/899P), was performed exactly as described previously (Lanciotti et al., 2007), the published assay for the detection of SINV via RT‐qPCR (Sane et al., 2012) was slightly modified. All in‐house and published but modified primers and probes, their sequences, positions on the corresponding genes, sizes of the amplicons, as well as reference molecules are listed in Table 1. Each RT‐qPCR based on TaqMan® chemistry was optimized for Quanta qScript XLT One‐Step RT‐qPCR ToughMix Kit (Quantabio, Beverly, MA, USA) with primer and probe concentrations of 0.5 μM each.

### 2.6 Blood meal analysis

In 2019, vertebrate hosts were identified from individual blood‐engorged female mosquitoes encountered in the questing traps as described previously (Camp et al., 2019). Briefly, DNA was extracted from homogenized abdomens using a commercial kit (DNeasy, Qiagen, Hilden, Germany) and subjected to PCR to amplify a region of the mitochondrial gene *16S rRNA* (Kitano et al., 2007) or *cytochrome b* (Cupp et al., 2004). The respective primers target vertebrate sequences but do not efficiently amplify invertebrate sequences of these gene regions. The resulting amplicons were sequenced by the Sanger method and compared to in‐house voucher sequences, or to publicly available sequences using the online version of BLASTn (Altschul et al., 1990).

### 2.7 Data collection of birds

In 2018, bird monitoring was conducted by driving (walking speed) along a road that encircles the airport (16 km), and counting the number and determining the species of the observed birds. In 2019, the scheme was changed to a ‘point stop’ method. At 19 locations around the airport's border, the number and the species of the encountered birds within a time period of five minutes were recorded. Bird counts were conducted twice each month, and the numbers averaged for each month.

In 2019, fresh bird carcasses from airplane strikes were collected and analysed for flaviviruses. The carcasses were frozen at −20°C for up to 1 month and thawed overnight at 4°C before dissection. Pooled organ sections (heart, liver, spleen, lung, and leg muscle) were homogenized in Dulbecco's phosphate‐buffered saline (DPBS, Gibco, Dublin, Ireland) using metal beads on a bead mill (TissueLyser II, Qiagen, Hilden, Germany), and RNA was extracted from the homogenate using a commercial kit (QIAamp Viral RNA Mini Kit, Qiagen, Hilden, Germany). The RNA was stored at −80°C until testing for flavivirus nucleic acid as described above.

## 3 RESULTS

### 3.1 Mosquito species

In total, 4850 mosquitoes were collected (2018: *n* = 2072, 2019: *n* = 2778); of these 4167 were females (2018: *n* = 1749, 2019: *n* = 2418) and 683 males (2018: *n* = 323, 2019: *n* = 360). Of the female mosquitoes, 4034 (96.8%) could be identified to species (or species complex) level. We caught mosquitoes from 17 different species of six genera (Table 2). Female *Cx. pipiens/torrentium* mosquitoes accounted for 93.5% (*n* = 1635) of the captured females in 2018, and 74.1% (*n* = 1792) of the captured females in 2019. A sample of females belonging to *Cx. pipiens/torrentium* (three arbitrarily chosen in 2018 and five selected by random sampling in 2019) were barcoded and identified as *Cx. pipiens* form *pipiens*. Further, we quite frequently captured females of *Aedes vexans* (2018: 38, 2019: 414), *Aedes sticticus* (2018: 2, 2019: 79), *Culiseta annulata* (2018: 2, 2019: 13), and *Cx. modestus* (2018: 10, 2019: 4) species.

**TABLE 2:** Species composition of the female (F) and male (M) mosquitoes caught at the airport. For males, only the genus level was determined

	2018		2019		
Species	F	M	F	M	Total
*Anopheles claviger*	–	–	2	–	2
*Anopheles hyrcanus*	–	–	1	–	1
*Anopheles maculipennis* complex	7	–	2	–	9
*Anopheles* sp.	–	1	2	1	4
*Aedes caspius*	2	–	7	–	9
*Aedes cinereus/geminus*	–	–	9	–	9
*Aedes geniculatus*	–	–	2	–	2
*Aedes japonicus*	1	–	–	–	1
*Aedes sticticus*	2	–	79	–	81
*Aedes vexans*	38	–	414	–	452
*Aedes* sp.	4	27	45	50	126
*Culex hortensis*	–	–	1	–	1
*Culex modestus*	10	–	4	–	14
*Culex pipiens/torrentium*	1635	–	1792	–	3427
*Culex territans*	1	–	–	–	1
*Culex* sp.	41	292	41	300	674
*Culiseta annulata*	2	–	13	–	15
*Culiseta longiareolata*	1	–	–	–	1
*Culiseta* sp.	–	1	–	9	10
*Coquillettidia richiardii*	3	–	3	–	6
*Uranotaenia unguiculata*	–	–	1	–	1
undefined	2	2	–	–	4
**Total**	**1749**	**323**	**2418**	**360**	**4850**

Abbreviation: sp., species.

### 3.2 Flaviviruses

**FIGURE 1:**
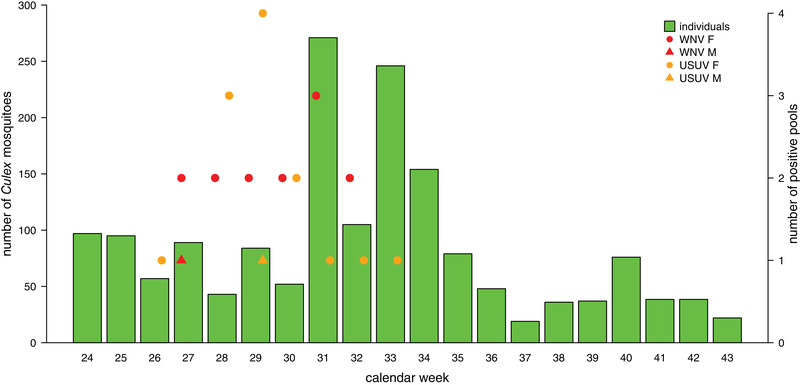
Number of collected mosquitoes of the genus *Culex* in 2018 and the corresponding number of positive virus pools

In total, 224 (2018) and 166 (2019) mosquito pools were tested for flaviviruses. In 2018, WNV nucleic acids were detected in 14 pools, and USUV nucleic acids were detected in 14 other pools, thus 28 flavivirus‐positive pools were detected in total (Table 3). These results were obtained by the respective virus‐specific RT‐qPCRs and confirmed by the universal flavivirus RT‐PCR with subsequent sequencing of all specific PCR products. Sequencing facilitated precise identification of the flaviviruses: all 14 WNVs belonged to lineage 2 and all 14 USUVs belonged to the Europe 2 cluster. Of these 28 positive pools, 26 consisted of female *Cx. pipiens/torrentium*, and two male *Culex* sp. mosquitoes (one among WNV‐pos. and one among USUV‐pos. pools). WNV‐positive pools were found from mid‐June to late July and USUV‐positive from mid‐June to early August (Figure 1, Table 3). Based on the detection of virus in at least 14 pools, the field MIR was calculated to be 6.8 (95% CI  =  [3.3, 10.4]) infected mosquitoes per 1000 mosquitoes for each virus.

**TABLE 3:** Characteristics of WNV‐ and USUV‐positive mosquito pools

Pool ID	Collection date	Mosquito species	Sex	No. of ind.	Ct value (specific RT‐qPCR)	Sequence length (bp)^a^	GenBank **acc**. no.
WNV lineage 2‐positive mosquito pools							
**AT‐109/18**	08.08.2018	*Cx. pip./torr*.	F	10	18.10	**799**	**MW160840**
AT‐117/18	08.08.2018	*Cx. pip./torr*.	F	10	24.48	830	MW160841
AT‐130/18	05.09.2018	*Cx. pip./torr*.	F	10	30.34	n.d.	–
AT‐136/18	05.09.2018	*Cx. pip./torr*.	F	7	34.58	n.d.	–
AT‐137/18	01.08.2018	*Cx. pip./torr*.	F	10	25.16	830	MW160842
AT‐140/18	01.08.2018	*Cx. pip./torr*.	F	10	34.09	n.d.	–
AT‐146/18	01.08.2018	*Cx*. sp.	M	2	30.08	n.d.	–
**AT‐158/18**	29.08.2018	*Cx. pip./torr*.	F	10	22.62	**812**	**MW160843**
AT‐160/18	29.08.2018	*Cx. pip./torr*.	F	10	23.46	830	MW160844
AT‐181/18	29.08.2018	*Cx. pip./torr*.	F	10	20.86	830	MW160845
AT‐187/18	16.08.2018	*Cx. pip./torr*.	F	10	28.45	830	MW160846
AT‐200/18	16.08.2018	*Cx. pip./torr*.	F	10	23.33	830	MW160847
AT‐261/18	22.08.2018	*Cx. pip./torr*.	F	10	21.01	830	MW160848
AT‐279/18	22.08.2018	*Cx. pip./torr*.	F	10	18.63	830	MW160849
USUV Europe 2‐positive mosquito pools							
**AT‐34/18**	18.07.2018	*Cx. pip./torr*.	F	10	31.64	**690**	**MW160850**
AT‐76/18	08.08.2018	*Cx. pip./torr*.	F	10	22.13	695	MW160851
AT‐107/18	08.08.2018	*Cx. pip./torr*.	F	10	21.90	695	MW160852
AT‐120/18	08.08.2018	*Cx. pip./torr*.	F	10	31.28	n.d.	–
AT‐127/18	05.09.2018	*Cx. pip./torr*.	F	10	30.68	695	MW160853
AT‐152/18	12.09.2018	*Cx. pip./torr*.	F	10	22.52	695	MW160854
**AT‐167/18**	29.08.2018	*Cx. pip./torr*.	F	10	17.90	**653**	**MW160855**
AT‐186/18	16.08.2018	*Cx. pip./torr*.	F	10	23.79	695	MW160856
AT‐190/18	16.08.2018	*Cx. pip./torr*.	F	10	24.92	695	MW160857
AT‐197/18	16.08.2018	*Cx*. sp.	M	10	30.62	695	MW160858
AT‐201/18	16.08.2018	*Cx. pip./torr*.	F	10	30.82	n.d.	–
AT‐202/18	16.08.2018	*Cx. pip./torr*.	F	10	24.81	695	MW160859
AT‐270/18	22.08.2018	*Cx. pip./torr*.	F	10	21.70	695	MW160860
AT‐280/18	22.08.2018	*Cx. pip./torr*.	F	2	24.34	695	MW160861

Abbreviations: *Cx*., *Culex*; *Cx. pip./torr., Cx. pipiens/torrentium*; ind., individuals; n.d., not detected;

^a^length without primer sequences.

Sequences with deletions in bold.

In total, amplification of specific PCR products and sequencing was possible for 10 out of 14 WNV‐positive pools and 12 out of 14 USUV‐positive pools. RT‐qPCR‐positive samples with Ct values > 30 (four WNV‐ and two USUV‐pos.) could not be detected by RT‐PCR (Table 3). Detailed sequence analysis found that, while eight of 10 obtained WNV sequences were 830‐bp long (length without primer sequences, from position 10147 to 10976 of the WNV reference sequence Goshawk/Hungary/2004, GenBank acc. no. DQ116961), the remaining two sequences exhibited deletions within the 3'UTR of 18 bp (positions 10501–10518) and 31 bp (positions 10480–10510), respectively (Table 3). Similarly for USUV, while 10 of 12 obtained sequences were 695‐bp long (length again without primer sequences, positions between 10106 and 10800 of the USUV reference sequence strain Vienna 2001, GenBank acc. no. AY453411), the remaining two sequences showed deletions within the 3'UTR of 5 bp (positions 10490–10494) and 41 bp (positions 10464–10504), respectively (Table 3).

The pairwise nucleotide sequence identities for the 10 newly established WNV sequences were calculated to be between 95.3% and 99.8%, corresponding to 1–39 (of 830) nucleotide differences between the sequences, with two non‐synonymous substitutions in the *NS5* open reading frames of two samples each (AT‐200/18, glutamic acid (E) instead of glycine (G) at position 3374, and AT‐158/18, isoleucine (I) instead of threonine (T) at position 3389, both compared to the WNV polyprotein reference sequence AAZ91684). The mean nucleotide difference for these 10 sequences was 7.35 nt (standard deviation = 2.5). The highest identities were determined for sequences AT‐137/18 and AT‐279/18, and the greatest differences were found between sequence AT‐109/18 and the two sequences AT‐117/18 and AT‐261/18. For the 12 USUV sequences, the pairwise sequence identities were between 93.3% and 100%, which corresponds to 0–46 (of 695) nucleotide differences. The highest identity rate was determined for sequences AT‐107/18 and AT‐280/18, and the lowest sequence similarity was found between the sequences AT‐167/18 and AT‐186/18. The mean nucleotide difference for these 12 sequences was 2.60 nt (standard deviation = 1.6), with a single non‐synonymous substitution in the *NS5* gene (AT‐167/18, proline (P) instead of leucine (L) at position 3418 compared to the USUV polyprotein reference sequence NC_006551).

**FIGURE 2:**
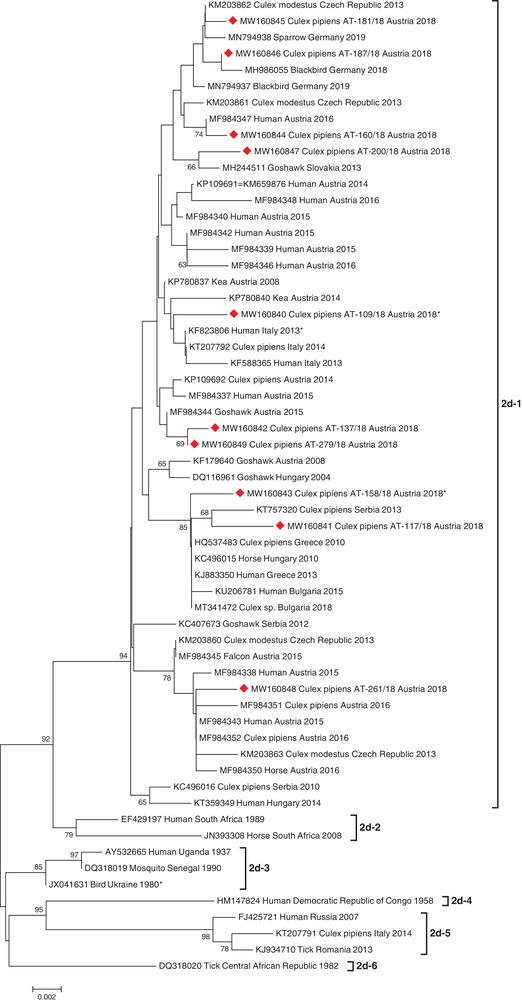
Phylogenetic tree demonstrating the genetic relationships among 60 WNV lineage 2 sequences. The 10 sequences generated in this study are indicated by red diamonds. Asterisks indicate sequences exhibiting deletions within the 3′UTR. Phylogenetic analysis was performed on 690‐bp long sequences within the *NS5*/3′UTR genomic region (positions 10147–10976 according to the WNV complete genome sequence, GenBank acc. no. DQ116961). For each sequence, the corresponding GenBank accession number, host species, country of origin, and collection year are indicated. Horizontal lines represent the genetic distances. Genetic sub‐lineages are indicated by vertical bars on the right. Bootstrap values above 60 are displayed at the nodes.

In addition to the sequence similarity analyses, the phylogenetic analysis confirmed the high diversity of WNV and USUV strains detected in this study. The WNV strains detected in this study belong to five sub‐clusters within the WNV sub‐lineage 2d‐1 (Figure 2). Specifically, four new mosquito sequences (AT‐181/18, AT‐187/18, AT‐160/18 and AT‐200/18) shared one sub‐cluster together with a human Austrian strain from 2016, four bird strains from Germany (2018–2019) and Slovakia (2013), and two mosquito strains from the Czech Republic (2013). The new sequence with the 31‐bp long deletion (AT‐109/18) occupied a second sub‐cluster together with WNV strains of two Austrian keas (2008 and 2014), and three sequences that originated from Italian humans and mosquitoes (2013–2014). Two further new sequences (AT‐137/18 and AT‐279/18) shared a third sub‐cluster with exclusively Austrian human, bird, and mosquito strains (2014–2015). The strain with the 18‐bp deletion (AT‐158/18) and another strain from our study (AT‐117/18), grouped in the fourth sub‐cluster, together with WNV sequences that originated from different species (human, horse, bird, and mosquito), countries (Austria, Hungary, Greece, Bulgaria, and Serbia), and years (from 2004 to 2018). In this sub‐cluster, the first European WNV strain (DQ116961, Goshawk, Hungary, 2004) and the first Austrian WNV strain (KF179640, Goshawk, 2008) are located. The last sequence (AT‐261/18) sub‐clustered together with six human, horse, bird, and mosquito sequences from Austria (2015–2016), two Czech mosquito strains from 2013, and one bird sequence from Serbia (2012).

**FIGURE 3:**
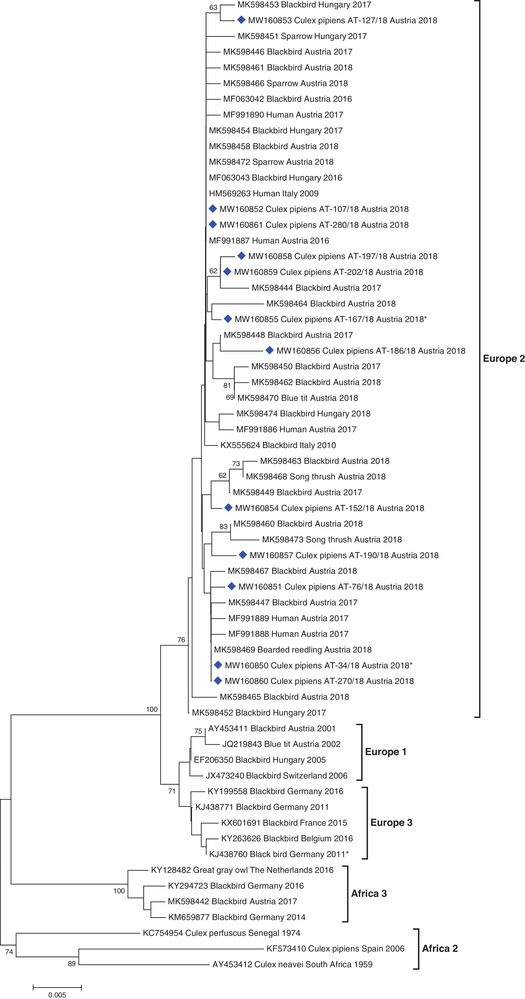
Phylogenetic tree demonstrating the genetic relationships among 62 USUV sequences. The 12 sequences generated in this study are indicated by blue diamonds. Asterisks indicate sequences exhibiting deletions within the 3′UTR. Phylogenetic analysis was performed on 695‐bp long sequences within the *NS5*/3′UTR genomic region (positions 10106–10800 according to the USUV complete genome sequence, GenBank acc. no. AY453411). For each sequence, the corresponding GenBank accession number, host species, country of origin, and collection year are indicated. Horizontal lines represent the genetic distances. Genetic lineages are indicated by vertical bars on the right. Bootstrap values above 60 are displayed at the nodes.

Similarly, all newly detected USUV strains were grouped in at least seven sub‐clusters among the USUV cluster Europe 2, together with other human and bird sequences from Austria, Hungary, and Italy, detected from 2009 to 2018 (Figure 3). Specifically, they showed a high relationship to sequences obtained from several Austrian blood donors and birds, both sampled during the mosquito seasons 2017 and 2018.

In 2019, all of the 166 sample pools were negative for WNV and USUV. However, in one pool of 14 male *Culex* sp., caught at the end of July, mosquito‐specific flaviviral RNA was detected by the universal flavivirus RT‐PCR. The 201‐bp long sequence exhibited 95.5% to 97.0% identity to Culex Iflavi‐like viruses 4 or Culex picorna‐like viruses, family *Iflaviridae*, which includes Deformed wing virus and Slow bee paralysis virus.

### 3.3 Other viral investigations

All mosquito pools collected in 2018 and 2019 tested negative for CVOV/BATV, CHIKV, TAHV and SINV by their corresponding RT‐qPCRs.

### 3.4 Blood meal analysis and bird monitoring

In 2019, we captured 23 female mosquitoes in which a previously taken blood meal was visible in the abdomen: 18 *Cx. pipiens/torrentium*, two *Cx*. sp. and three *Ae. vexans*. Hosts of *Cx. pipiens* complex females were identified as *Pica pica* (22.2%), *Lepus europaeus* (11.1%), and one blood meal came each from *Capreolus capreolus, Coturnix coturnix, Falco tinnunculus*, *Phasianus colchicus* and *Sturnus vulgaris* (5.6% each). Thus, 44.4% of the blood meals were from birds, and 16.7% from mammals, whereas 38.9% of the *Cx. pipiens/torrentium* blood meals could not be identified. The blood meals of the two *Cx*. sp. could not be identified either. In *Ae. vexans*, the blood meals originated from *Lepus europaeus* (66.7%) and *Capreolus capreolus* (33.3%).

**FIGURE 4:**
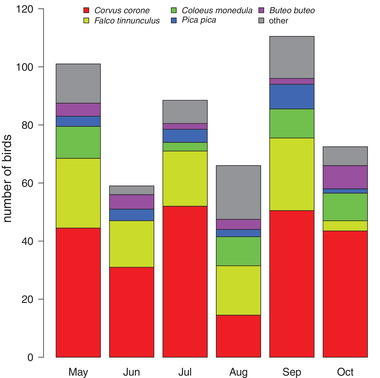
Observed birds at the airport (averaged from 2018 and 2019) during the mosquito‐monitoring period from May to October

During the bird monitoring at the airport, 11 species were recorded (Figure 4); the most common were crows (*Corvus corone*) and kestrels (*Falco tinnunculus*). Eurasian magpies (*Pica pica)*, the most common host for the identified *Cx. pipiens/torrentium* blood meals, accounted only for 1.5–8.5% of the bird counts from May to October. Flaviviral nucleic acids were not detected in any of the tested bird tissues taken from carcasses following airplane strikes.

## 4 DISCUSSION

During two years of monitoring at the airport, we detected an unusually high number of WNV‐ and USUV‐positive mosquito pools only in the first year, 2018. Although we detected only endemic WNV and USUV strains, the partial genomic sequence of each mosquito pool‐derived virus was unique. We collected no exotic mosquito species, except a single specimen of *Ae. japonicus*. The observed mosquito population was comprised largely of *Cx. pipiens/torrentium*, which is common in heavily built‐up areas such as airports (Ibañez‐Justicia et al., 2017) or other urban environments in central Europe (Krüger et al., 2014; Lebl et al., 2015) and North America (Pecoraro et al., 2007; Trawinski & MacKay, 2010). A closer examination of sampled individuals showed that they belonged to *Cx. pipiens* f. *pipiens*, which seems to be the predominant variant of this species complex in eastern Austria (Zittra et al., 2016). *Culex pipiens* mosquitoes are known to be competent vectors of several arboviruses, including those endemic to Europe (WNV and USUV), as well as St. Louis encephalitis virus, SINV, and Rift Valley fever virus (Turell, 2012). Otherwise, the mosquito community at the airport was similar to other mosquito surveys in nearby urban environments in Austria (Lebl et al., 2015).

Although both WNV and USUV have been established in Austrian mosquito populations for many years (Chvala et al., 2007; Weissenböck et al., 2003; Wodak et al., 2011), only targeted vector surveys at sites of confirmed human, horse, or bird WNV cases (de Heus et al., 2020; Kolodziejek et al., 2015, 2018), or of USUV‐associated bird deaths (Camp et al., 2019), have provided positive detection of virus in the mosquito population. Routine nationally or regionally organized mosquito monitoring efforts in Austria rarely detect arboviruses in mosquito pools (including a nationwide monitoring survey during 2018). In nearly all of these cited cases, WNV and USUV were routinely detected in *Cx. pipiens/torrentium* mosquito pools, and it is therefore not surprising that arboviral RNA was detected only in pools of this complex in our study. The presence of WNV and USUV nucleic acids in pools of male *Culex* sp. mosquitoes most likely indicates vertical transmission. Infected female *Cx. pipiens* mosquitoes have been shown in both, field and laboratory experiments, to transmit WNV to their progeny, where the virus may persist trans‐stadially resulting in infectious adult mosquitoes (Nelms et al., 2013; Reisen et al., 2006). As vertical transmission has been demonstrated to occur at a relatively low rate (Anderson et al., 2008), the finding of WNV and USUV in two pools of male *Culex* sp. mosquitoes supports the increased activity of WNV and USUV in the sampled area. However, we acknowledge that the detection of arboviral nucleic acids in individual or pools of mosquitoes does not prove infection.

The results of the blood meal analysis, with a high proportion of *Cx. pipiens/torrentium* females feeding on birds, are in line with previous studies showing that *Cx. pipiens* mosquitoes are ornithophilic, but occasionally feed on mammals, reptiles, or amphibians as well (Muñoz et al., 2012; Radrova et al., 2013; Rizzoli et al., 2015; Roiz et al., 2012). Although they were encountered rarely during the bird counting events, Eurasian magpies (*Pica pica*) were the preferred hosts of *Cx. pipiens/torrentium* females based on the blood meal analysis. These results match those of Rizzoli et al. (2015), who demonstrated that, in Europe, magpies and blackbirds (*Turdus merula*; not recorded at the airport) were significantly preferred by *Cx. pipiens*. Although we did not calculate a preference index, magpies were not the most abundant birds at the airport, as we encountered carrion/hooded crows (*Corvus corone* ssp.) and common kestrels (*Falco tinnunculus*) more frequently (Figure 4). However, only one of 18 blood meals from *Cx. pipiens/torrentium* was identified as a common kestrel. As magpies have been shown to be highly susceptible to WNV infections, they could thus be an important factor in the transmission cycle of this virus (Jiménez de Oya et al., 2018; Napp et al., 2019). Of note, it is possible that the high proportion of blood from magpies relative to their abundance represents a sampling bias of our study, as we found a magpie nest in a courtyard near where the mosquito sampling was performed.

Although *Cx. pipiens/torrentium* mosquitoes clearly prefer avian hosts, flaviviruses were not detected in any of the bird carcasses collected in this area during the study period. This could be due to a sampling bias, as no magpies were among the tested carcasses, which also reflects their infrequent encounters during the ornithological survey. Other bird species observed during the study, from which carcasses were tested for virus, are known to support relatively high viremia following WNV infection, particularly the corvids and birds of prey (Komar et al., 2003; Work et al., 1955). However, the unexpectedly high infection rate of mosquitoes trapped at the international airport in 2018 is in accordance with the reported, relatively high numbers of WNV and/or USUV infections in humans (Aberle et al., 2018), horses (de Heus et al., 2020), and birds (Weidinger et al., 2020) in Austria in 2018.

In Europe, 2018 has been widely recognized as a year with a record‐breaking incidence of WNV cases in both, humans and horses (Camp & Nowotny, 2020). In Austria, the number of human WNV cases in 2018 was 4‐ to 5‐fold higher than in previous and following years (2018, *n* = 20; 2016 and 2017, *n* = 5 each; 2019 *n* = 4). All but two cases occurred in the City of Vienna and the directly adjacent regions (ECDC, 2018, 2019). It is therefore not surprising that we also report a relatively high MIR for WNV in 2018 at our study site. In general, a MIR  >1 seems to be associated with an increased incidence of human WNV cases, compared to longitudinal surveys with similar sampling methods in the neighbouring country of Italy (Calzolari et al., 2015, 2020; Cerutti et al., 2012), although strict comparisons of MIR between surveillance programmes may not be reliable (Chakraborty & Smith, 2019). Moreover, we note that the incidence of WNV in humans in Austria is much lower than in Italy, in both epidemic and non‐epidemic years (Aberle et al., 2018; ECDC, 2018, 2019). Increased WNV activity in 2018 was likely due to specific environmental conditions that promoted mosquito abundance and increased the likelihood of early‐season encounters between infected mosquitoes and suitable competent vertebrate reservoirs (Camp & Nowotny, 2020). It remains to be seen why WNV cases in Europe returned to baseline in 2019, given the similar meteorological conditions. We did not record meteorological variables in this study, but we monitored both the mosquito and avian communities. In our analysis, we averaged bird counts at the airport from 2018 and 2019 (Figure 4), as there was no difference in a given species’ abundance between the years. As has been noted by others (reviewed in Camp & Nowotny, 2020), the observed decline of infections in 2019 may be due to increased flock immunity of avian amplifying hosts, acquired during the 2018 epizootic/epidemic year. However, we did not test the serological status of the bird carcasses that we investigated. Because USUV utilizes similar competent mosquito vectors as WNV (primarily *Cx. pipiens*; Fros et al., 2015), and probably similar vertebrate amplifying hosts, with blackbirds being notable for their high mortality rate associated with USUV infection (Chvala et al., 2007; Weidinger et al., 2020; Weissenböck et al., 2002), it is not surprising that we detected the same high MIR for USUV as for WNV in 2018, but found no infections in 2019.

The relatively high prevalence of WNV and USUV in 2018 was similarly experienced throughout Europe, and was likely due to environmental factors which led to increased mosquito abundance and increased transmission efficiency in the early season (Camp & Nowotny, 2020). However, it is unclear what factors were responsible for the unexpectedly high heterogeneity of WNV and USUV strains detected in *Cx. pipiens/torrentium* mosquitoes trapped at this small courtyard of the international airport. Continued surveillance, including ‘control’ sites in other urban and peri‐urban environments, will indicate whether the airport is useful for monitoring WNV activity on a larger (regional) scale. Our results support the observation of others that importation of exotic mosquitoes and/or viruses via airports is probably a rare event in Europe, but nonetheless such a surveillance is effective in the early detection of exotic species (Ibanez‐Justicia et al., 2020). Our results clearly illustrate the focal nature of arbovirus activity, with possible intense episodic transmission activity (such as in 2018), and support the value of targeted surveillance to monitor WNV and USUV activity. Furthermore, we show that ecological habitats at airports can support the transmission of WNV and USUV, thereby increasing the risk of propagation of mosquitoes and/or viruses to non‐endemic regions. Our observations also highlight the importance of monitoring arthropod vectors at sites of international transport, where the potential for intense arbovirus activity clearly exists, and suggest that strict vector control/disinsection protocols should be followed at these sites.

We detected a relatively high sequence diversity of WNV (an average of 7.35 nt over the sequence, or 8.86 nt per 1000 sites) compared to USUV (an average of 2.60 nt over the sequence or 3.74 nt per 1000 sites). This diversity was indeed higher than, for example, an analysis of Italian WNV lineage 2 isolates over eight years (mean 1.73 nt per 1000 sites) (Veo et al., 2019). Phylogenetically, these mosquito‐derived WNV strains are represented in almost all sub‐clusters within‐cluster 2d‐1 (Figure 2). They cluster together with other sequences independent of species (human, horse, bird, mosquito), geographic region (nine European countries) or collection year (2004–2018), further emphasizing their high diversity. We observed no special association with previously published Austrian strains, although two of the analysed WNV sequences belonged to an exclusively Austrian sub‐cluster (Kolodziejek et al., 2018).

The mosquito‐derived USUV strains identified here were distributed widely over the phylogenetic tree within the cluster Europe 2 (Figure 3). They are highly related to Austrian human sequences from 2017 (Bakonyi et al., 2017), as well as to Austrian and Hungarian bird sequences from 2017 and 2018 (Weidinger et al., 2020). Sequencing of Austrian human USUV strains detected in 2018 (Aberle et al., 2018) was performed in another genomic region; therefore, a direct phylogenetic comparison with the current mosquito strains was not possible. However, upon closer examination of joint sequences, at least three mosquito strains would most probably also cluster together with the majority of USUV strains generated from Austrian blood donors in 2018. Others have noted the comparatively low genetic variability of USUV within the established genetic lineages circulating across Europe, particularly since the spread of the Europe 2 lineage beginning in 2015/2016 (Bakonyi et al., 2017; Weidinger et al., 2020), and even over broader geographic areas (Nikolay et al., 2013). Thus, our data represent an interesting aspect of the co‐circulation of USUV and WNV that has yet to be addressed. Namely, albeit the arthropod vector population and host availability are presumably the same for both viruses, we observed a much higher mutation rate for WNV than for USUV. There are likely many factors that may contribute to this, which should be investigated more thoroughly in future studies.

The uniqueness of the WNV and USUV strains we identified is additionally underlined by deletions of different lengths within the 3′UTRs in two WNV and two USUV strains (Figures 2 and 3, correspondent sequences are indicated with asterisks). While short deletions did not seem to have any influence on phylogenetic clustering of the correspondent virus strain (see Figure 3, seq. AT‐34/18), the sequences with longer deletions build rather separate clades (see Figure 2, seq. AT‐109/18 and AT‐158/18). Since the emergence of USUV in Austria in 2001 (Weissenböck et al., 2002), and Hungary in 2005 (Bakonyi et al., 2007), as well as the emergence of WNV in Hungary in 2004 and in Austria in 2008 (Bakonyi et al., 2006, 2013; Wodak et al., 2011), we have investigated and genetically characterized several hundred virus strains from humans, horses, birds, and mosquitoes, but we have never before observed such deletions to date. Deletions in 3'UTRs of WNV and USUV sequences are also rare worldwide. They were only observed in two WNV sequences: KF823806 (human, Italy, 2013, unpublished, see Figure 2, sub‐cluster 2d‐1, next to seq. AT‐109/18) and JX041631 (bird, Ukraine, 1980, unpublished, see Figure 2, sub‐cluster 2d‐3), as well as in one European USUV sequence: KJ438760 (33‐bp deletion, black bird, Germany, 2011; Engel et al., 2016; see Figure 3, cluster Europe 3). Engel et al. (2016) additionally discovered deletions in other genomic regions of two African USUV strains: ARB1803 (GenBank acc. no. KC754958) and HB81P08 (GenBank acc. no. KC754955). It has been suggested for another mosquito‐borne flavivirus, namely DENV, that hypervariability of the 3′UTR could affect the replication of this virus during different vector/host transmission cycles (Alvarez et al., 2005; Shurtleff et al., 2001), although we note that the deletions we observed were apparently not in known functional domains of the 3′UTR (Robyn et al., 2014). Perhaps the particular ecological circumstances in our study, namely intense transmission activity at a small location likely involving few vertebrate hosts (i.e., a nesting magpie), may direct future studies of the significance of such deletions in flaviviruses.

## 5 CONCLUSIONS

We report the establishment of a mosquito monitoring programme at a large international airport in Central Europe, to augment the national surveillance programme. The first year of monitoring coincided with the largest outbreak of WNV in Europe on record, and our observations reflect the high transmission activity of both WNV and USUV in 2018. Combining bird surveys and mosquito blood meal analyses to identify hosts provided additional information about the transmission patterns of WNV and USUV at the airport, both of which likely involve certain avian hosts (especially the Eurasian magpie, *Pica pica*) and mosquito species (*Cx. pipiens/torrentium)* for virus maintenance, amplification and spillover. Therefore, mosquito control efforts at airports should consider targeting these species specifically.

## CONFLICT OF INTEREST

The authors declare no conflict of interest.

## ETHICAL STATEMENT

Not applicable because in Lower Austria the collection of unprotected insect species (as mosquitoes) is only subject to a permit if larger quantities are removed or the collection is for commercial purposes. Further, only dead birds (bird strikes) were collected and analysed in the study.

## Data Availability

All WNV and USUV sequences generated in this study are openly available at the NCBI database (https://www.ncbi.nlm.nih.gov) with the following accession numbers: MW160840‐MW160849 and MW160850‐MW160861, respectively.
